# Increased circulation of GII.17 noroviruses, six European countries and the United States, 2023 to 2024

**DOI:** 10.2807/1560-7917.ES.2024.29.39.2400625

**Published:** 2024-09-26

**Authors:** Preeti Chhabra, Shan Wong, Sandra Niendorf, Ingeborg Lederer, Harry Vennema, Mirko Faber, Athinna Nisavanh, Sonja Jacobsen, Rachel Williams, Aoife Colgan, Zoe Yandle, Patricia Garvey, Haider Al-Hello, Katia Ambert-Balay, Leslie Barclay, Miranda de Graaf, Cristina Celma, Judith Breuer, Jan Vinjé, Amy Douglas

**Affiliations:** 1 Division of Viral Diseases, Centers for Disease Control and Prevention, Atlanta, United States; 2 Enteric Virus Unit, UK Health Security Agency, London, United Kingdom; 3 Consultant Laboratory for Norovirus, Department of Infectious Diseases, Robert Koch Institute, Berlin, Germany; 4 Austrian Agency for Health and Food Safety, Graz, Austria; 5 National Institute for Public Health and the Environment (RIVM), Bilthoven, the Netherlands; 6 Department of Infectious Disease Epidemiology, Robert Koch Institute, Berlin, Germany; 7 French Public Health Agency, Santé Publique France, Saint-Maurice, France; 8 University College London Great Ormond Street Institute of Child Health, London, United Kingdom; 9 Gastroenteric, Zoonotic and Vectorborne Diseases Team, HSE-Health Protection Surveillance Centre, Dublin, Ireland; 10 UCD National Virus Reference Laboratory, University College Dublin, Belfield, Dublin, Ireland; 11 Microbiology Unit, Department of Public Health, National Institute for Health and Welfare, Helsinki, Finland; 12 French National Reference Centre for Gastroenteritis Viruses, Virology Laboratory, University Hospital of Dijon, Dijon, France; 13 Department of Viroscience, Erasmus MC, Rotterdam, the Netherlands; 14 Gastrointestinal Infections, Food Safety and One Health Division, UK Health Security Agency, London, United Kingdom

**Keywords:** Norovirus, GII.17, emergence, GII.4, gastroenteritis

## Abstract

We report an increase in GII.17 norovirus outbreaks and sporadic infections of acute gastroenteritis in Austria, Germany, France, Ireland, the Netherlands, England and the United States during the 2023/24 season. A decrease in GII.4 coincided with GII.17 prevalence increasing to between 17% and 64% of all GII detections. Overall, 84% of the GII.17 strains clustered closely with strains first reported in Romania in 2021 and two new sub-lineages were identified. Norovirus surveillance and molecular characterisation should be prioritised this winter.

During the 2023/24 norovirus season (July 2023 to June 2024), elevated norovirus activity with an increase of GII.17 noroviruses was reported in at least six European countries, including Austria, France, Germany, Ireland, the Netherlands and England [1]. A similar trend was observed in the United States (US), where the proportion of GII.17 outbreaks exceeded the number of GII.4 outbreaks [2]. Given the sudden increase in detections of GII.17 noroviruses and their rapid geographic spread, we provide an update describing the prevalence and characterisation of this norovirus strain in known affected countries.

## Increase in GII.17 norovirus detections

To investigate this sudden rise in GII.17 norovirus activity, norovirus detection and genotyping data from 2019 to 2024 were provided by public health agencies of the six aforementioned countries and Finland (epidemiological data were shared with the United Kingdom Health Security Agency and genomic data were shared with the US Centers for Disease Control and Prevention). Depending on the established surveillance system in each country, data were available at outbreak level or for individual cases, all were deduplicated and grouped into seasons from July to June of each year. The percentage of samples typed as GII.17 or GII.4 was calculated by country and season, then aggregated into non-pandemic and pandemic three-season averages (non-pandemic: 2017/18, 2018/19 and 2022/23, pandemic: 2019/20, 2020/21 and 2021/22) to exclude the period impacted by the COVID-19 pandemic and allow comparison with the 2023/24 season.

**Figure 1:**
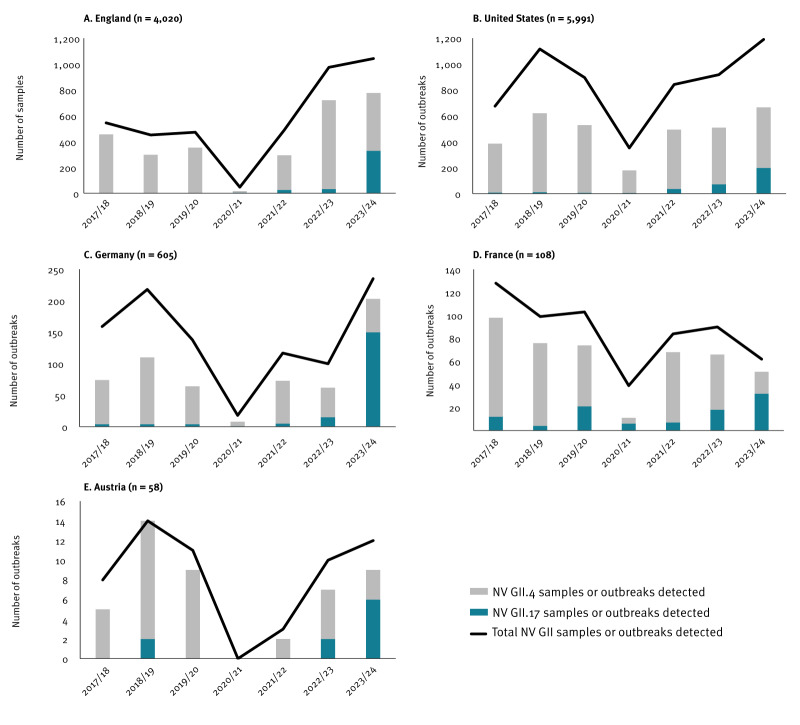
Trends of GII norovirus samples or outbreaks typed as genotype GII.17 or GII.4 by season, four European countries and the United States, 2017–2024 (n = 10,782)

**Figure 2:**
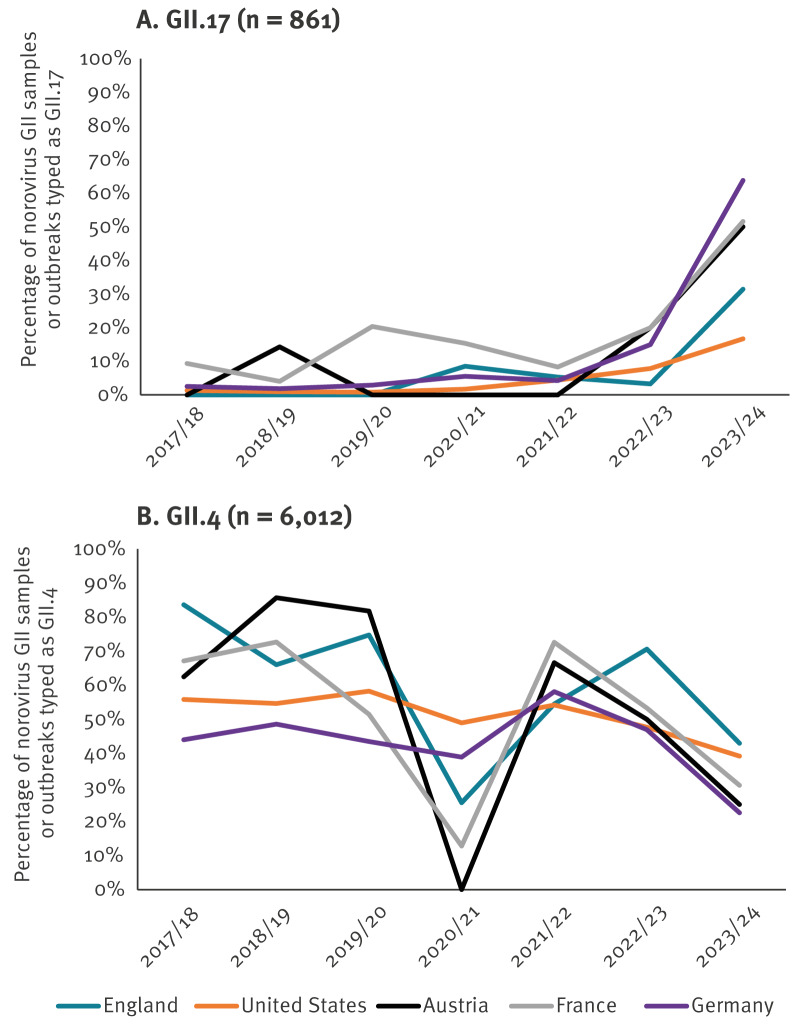
Percentage of GII norovirus outbreaks or samples typed as genotype GII.17 or GII.4 by season, four European countries and the United States, 2017–2024 (n = 6,873)

In all five countries providing epidemiological data (England, the United States, Germany, France and Austria) before the 2023/24 season, GII.4 was the most commonly detected genotype (Figure 1) accounting for over 50% of infections per season, with GII.17 accounting for less than 15%. Over the study period, detections of GII.17 transitioned from sporadic to comprising between 17% and 64% of all GII cases/outbreaks typed during the 2023/24 season (Figure 2), representing at least a fourfold increase compared with the non-pandemic three-season average in all countries (Table). Simultaneously, a notable decrease in the number of GII.4 cases/outbreaks was observed, although this was less pronounced in the US. GII.17 detection has increased in the colder months in France since 2019/20, in the US since 2021/22 and only in Austria and England during 2024. In early 2024, Finland reported higher than usual norovirus activity [3] and while historically they have sporadically detected GII.17, they reported no GII.4 or GII.17 detections in the 2023/24 season. Although Ireland suspended norovirus typing from 2019 to 2023, on resumption in 2024, GII.17 was detected for the first time and continues to increase in prevalence (data not shown). Stratification of English 2023/24 data by age group and month shows that most samples were from adults aged ≥ 65 years, but since March 2024, there have been more norovirus GII.17 than GII.4 cases in children aged 11–17 years in England. Clinical data were insufficient to assess whether there is a difference in clinical severity between these genotypes.

NV: norovirus.

The decrease in samples or outbreaks typed across all countries during the 2020/21 season coincides with the COVID-19 pandemic and the control measures implemented, which varied by country and duration, but likely impacted both quality of surveillance and incidence of norovirus. Since these impacts also extend back into the latter part of the 2019/20 season and into the 2021/22 season, these three seasons are defined as ‘the pandemic period’.

Austria, France, United States and Germany provided data at outbreak level, whereas England provided data at the individual sample level.

The decrease in samples or outbreaks typed as GII.4 across all countries coincides with the COVID-19 pandemic and the control measures implemented, which varied by country and duration, but likely impacted both quality of surveillance and incidence of norovirus. Since these impacts also extend back into the latter part of the 2019/20 season and into the 2021/22 season, these three seasons are defined as ‘the pandemic period’.

Austria, France, United States and Germany provided data at outbreak level, whereas England provided data at the individual sample level.

**Table:** Percentage of norovirus GII outbreaks or samples typed as genotypes GII.17 and GII.4, four European countries and the United States, 2017–2024 (n = 6,873)

Time period	GII.17					GII.4				
	England	US	Germany	Austria	France	England	US	Germany	Austria	France
Non-pandemic 3-season average^a^	1%	3%	6%	11%	11%	73%	53%	47%	66%	64%
COVID-19 pandemic 3-season average^b^	5%	2%	4%	0%	15%	52%	54%	47%	49%	46%
2023/24 season	31%	17%	64%	50%	52%	43%	39%	23%	25%	31%
Difference between non-pandemic average^a^ and 2023/24 season	30%	14%	58%	39%	39%	−30%	−14%	−24%	−41%	−33%

US: United States.

^a^ Non-pandemic three-season average calculated from the three seasons of 2017/18, 2018/19 and 2022/23, before and after the COVID-19 pandemic.

^b^ Pandemic three-season average calculated from the three seasons of 2019/20, 2020/21 and 2021/22, during which surveillance and incidence of norovirus were likely impacted to varying degrees in each country.

## Phylogenetic analyses 

**Figure 3:**
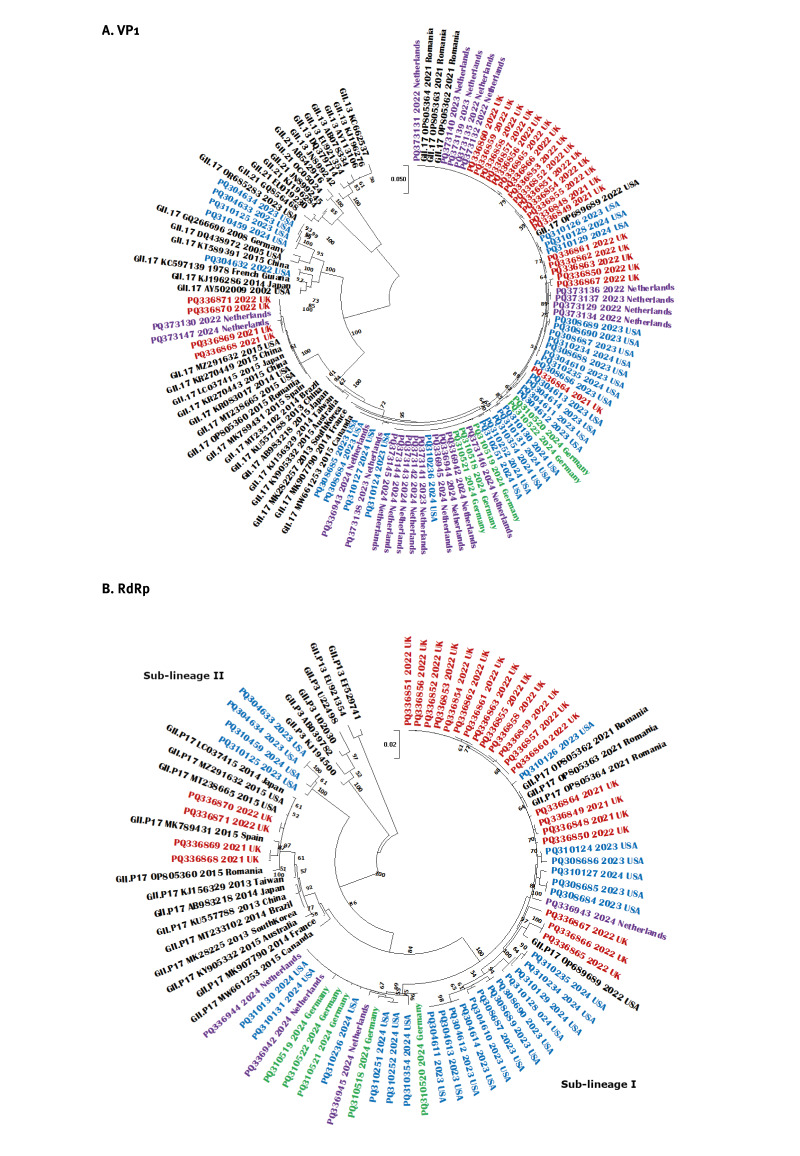
Phylogenetic analyses of GII.17 strains circulating in three European countries and the United States, 2021–2024 (n = 107)

VP1: Major capsid protein VP1; RdRp: RNA-dependent RNA polymerase.

For phylogenetic analyses, length and genomic region of sequences that were available for analyses varied markedly between countries since each country has different surveillance systems to capture norovirus activity. We received 63 complete genomes (Germany, n = 5; the Netherlands, n = 4; US, n = 30; England, n = 24), 18 complete VP1 (the Netherlands) and 76 partial GII.17 sequences (VP1 region C or P2 region and RNA-dependent RNA polymerase (RdRp) 320 nucleotides: Germany, n = 14; Austria, n = 43; Ireland, n = 19) from 2021 to 2024. Phylogenetic analyses of complete VP1 sequences (530 amino acids) and RdRp sequences (760 nucleotides from 3’ end of ORF1) from complete genomes confirmed the genotype of 2023/24 strains as GII.17[P17] (Figure 3). 

Maximum-likelihood phylogenetic trees were computed within MEGA11 [18] using (A) the Jones-Taylor-Thornton [19] (amino acid) and (B) Tamura-Nei [20] (nucleotide) models with gamma distribution of evolutionary rates among sites and 100 bootstrap replications.

GenBank numbers are listed with each sequence; known GII.17 variants were identified using the prototype sequences (n = 26, black) [7] and sequences from each country are shown in different colours: England (n = 24, red), United States (n = 30, blue), Germany (n = 5, green) and the Netherlands (n = 22, purple).

Of the 63 complete genome sequences, 53 (84%) clustered closely with GII.17 Romania-2021 strains (OP805362) [4] in both VP1 and RdRp regions suggesting a possible origin. All complete genomes were submitted to GenBank (accession numbers are provided in Supplementary Table S1). All Romania-2021-like strains were genetically closer to strains from France (MK907790) and Canada (MW661253) from the 2014/15 season for VP1 (Figure 3A). Six (10%) England strains clustered with the Kawasaki 308 (AB983218) cluster that first emerged in 2014/15 while five (8%) 2023 strains from the US clustered with GII.17 strains detected as early as 1978 and 2004/05 (Figure 3A). In the RdRp region, GII.P17 2023/24 strains formed two new sub-lineages (Figure 3B). Most (84%) sequences formed new sub-lineage I and were closer to Romania-2021 strains. Four US strains formed a new sub-lineage II between GII.P17 and GII.P3 strains (Figure 3B). Supplementary Figure S1 provides 2xSD analyses, which ruled out the possibility of a new P-genotype. Interestingly, all US sequences had a P31 P-type as summarised in Supplementary Table S1, along with strain ID and GenBank number. Supplementary Figures S2, S3 and S4 provide the phylogenetic analyses for partial sequences from Austria, Ireland and Germany confirmed the GII.17[P17] genotype. The spread of these new sub-lineages warrants ongoing monitoring.

## Discussion

Human noroviruses are the leading cause of acute gastroenteritis (AGE) worldwide in all age groups, causing an estimated 136,000 to 278,000 deaths annually [5,6]. Based on the amino acid diversity of the major capsid protein VP1, noroviruses are divided into 10 genogroups (GI to GX) [7]. Of these, viruses from five genogroups (GI, GII, GIV, GVIII and GIX) infect humans but the majority of the norovirus infections are associated with GI (9 genotypes) and GII (23 genotypes) viruses [7-10]. Since 2012, GII.4 Sydney viruses have been responsible for more than half of sporadic cases and outbreaks globally [3,8,11]. In addition to capsid-based genotyping, noroviruses can also be classified based on their RdRp-type (P-type) nucleotide sequences and at least 60 P-types have been recognised [7].

In 2021, a large GII.17 outbreak was reported in Romania [4] with similar strains from sporadic cases in Nizhny Novgorod, Russia (GenBank: OP712199) and from contaminated oyster-related AGE outbreaks in Washington and California (GenBank: OP689689) in 2022. During the 2023/24 season, six European countries and the US observed a notable increase in GII.17 norovirus infections that were genetically similar to the Romania-2021 strains. Since early 2024, these Romania-2021-like strains have been detected more frequently and in a more diverse range of age groups than GII.4 in most countries, however there is limited information on whether there is any difference in severity of illness associated with GII.17 compared with GII.4.

GII.17 viruses have been circulating at low levels for many years along with the predominant GII.4 viruses, except in 2014/15 when the GII.17 Kawasaki 308 (GII.17[P17]) strain emerged and spread globally [12,13]. This rise in GII.17 in 2014/15 coincided with a decline in GII.4 Sydney[P31] infections and the emergence of a recombinant GII.4 Sydney[P16] strain. In 2016, this GII.4 Sydney[P16] strain surpassed both GII.4 Sydney[P31] and, in several East Asian countries, GII.17[P17] and became the predominant norovirus genotype worldwide [8,13-16]. Interestingly, in 2023/24 after ca 8 years of GII.4 Sydney[P16] predominance, an increase of GII.17 infections has now been observed again. In England, the GII.17 increase has persisted into the start of the 2024/25 season, with GII.17 accounting for 77% of typed strains [17].

Since 1978, GII.17 viruses with 5 different P-types (GII.P17, GII.P13, GII.P16, GII.P3 and GII.P4) have been reported [13], demonstrating their capacity to easily switch polymerases through recombination. While in the 2023/24 season, the VP1 amino acid sequences showed minimal changes compared with 2014/16 GII.17 strains, their RdRp sequences form two new sub-lineages within the GII.P17 P-type cluster. The capacity of GII.17 viruses to switch their ORF1 (P-type) and to create new sub-lineages might have helped these viruses to persist in the population over 4 decades.

## Conclusion

Our findings aim to raise awareness about an increase of the number of GII.17 norovirus infections in Europe and the US during the 2023/24 season. We determined the majority of the VP1 and RdRp sequences as Romania-2021 like GII.17[P17] strains, with two new sub-lineages of GII.P17 identified. Further work to assess their spread and determine any difference in clinical severity or impact should be prioritised alongside molecular characterisation heading into the winter months.

## Supplementary Data

384000 Supplement
