# Supplementary material for: Increased circulation of GII.17 noroviruses, six European countries and the United States, 2023 to 2024
Source: Euro Surveill. 2024 Sep 26;29(39):2400625. doi: 10.2807/1560-7917.ES.2024.29.39.2400625 (PMC11484341; doi:10.2807/1560-7917.ES.2024.29.39.2400625)

Supplementary material

This supplementary material is hosted by Eurosurveillance as supporting information alongside the article "Increased circulation of GII.17 noroviruses, six European countries and the United States, 2023 to 2024", on behalf of the authors, who remain responsible for the accuracy and appropriateness of the content. The same standards for ethics, copyright, attributions and permissions as for the article apply. Supplements are not edited by Eurosurveillance and the journal is not responsible for the maintenance of any links or email addresses provided therein

Table S1: GenBank accession numbers of the GII.17 complete genome sequences from season 2023/24.

GenBank accession number	Strain ID	Genotype	Year	Country
PQ304632	SanMateo0237	GII.17[P31]	2022	USA
PQ304633	SanFrancisco0147	GII.17[P17]	2023	USA
PQ304634	SanFrancisco0148	GII.17[P17]	2023	USA
PQ304610	Michigan0047	GII.17[P17]	2023	USA
PQ304611	Clinton0099	GII.17[P17]	2023	USA
PQ304612	Clinton0100	GII.17[P17]	2023	USA
PQ304613	Clinton0102	GII.17[P17]	2023	USA
PQ304614	Clinton0104	GII.17[P17]	2023	USA
PQ310354	Boone0006	GII.17[P17]	2024	USA
PQ310251	NewYork0002	GII.17[P17]	2024	USA
PQ310252	NewYork0004	GII.17[P17]	2024	USA
PQ308684	Maimi0014	GII.17[P17]	2023	USA
PQ308685	Maimi0015	GII.17[P17]	2023	USA
PQ308686	Franklin0020	GII.17[P17]	2023	USA
PQ308687	Fairfield0028	GII.17[P17]	2023	USA
PQ308688	Fairfield0029	GII.17[P17]	2023	USA
PQ308689	Franklin0095	GII.17[P17]	2023	USA
PQ308690	Franklin0097	GII.17[P17]	2023	USA
PQ310124	Oregon7046	GII.17[P17]	2023	USA
PQ310125	Oregon7112	GII.17[P17]	2023	USA
PQ310126	Oregon8004	GII.17[P17]	2023	USA
PQ310127	Oregon8010	GII.17[P17]	2024	USA
PQ310128	Oregon8013	GII.17[P17]	2024	USA
PQ310129	Oregon8015	GII.17[P17]	2024	USA
PQ310130	Oregon8026	GII.17[P17]	2024	USA
PQ310131	Oregon8031	GII.17[P17]	2024	USA
PQ310459	Sheboygan0502	GII.17[P17]	2024	USA
PQ310234	Park0001	GII.17[P17]	2024	USA
PQ310235	Park0002	GII.17[P17]	2024	USA
PQ310236	Wyoming0005	GII.17[P17]	2024	USA
PQ310518	DE019	GII.17[P17]	2024	Germany

PQ310519	DE020	GII.17[P17]	2024	Germany
PQ310520	DE021	GII.17[P17]	2024	Germany
PQ310521	DE022	GII.17[P17]	2024	Germany
PQ310522	DE023	GII.17[P17]	2024	Germany
PQ336847	WT-NORO-0787	GII.17[P17]	2016	UK
PQ336848	WT-NORO-1934	GII.17[P17]	2021	UK
PQ336849	WT-NORO-2019	GII.17[P17]	2021	UK
PQ336850	WT-NORO-2117	GII.17[P17]	2022	UK
PQ336851	WT-NORO-2269	GII.17[P17]	2022	UK
PQ336852	WT-NORO-2270	GII.17[P17]	2022	UK
PQ336853	WT-NORO-2315	GII.17[P17]	2022	UK
PQ336854	WT-NORO-2317	GII.17[P17]	2022	UK
PQ336855	WT-NORO-2148	GII.17[P17]	2022	UK
PQ336856	WT-NORO-2163	GII.17[P17]	2022	UK
PQ336857	WT-NORO-2316	GII.17[P17]	2022	UK
PQ336858	WT-NORO-2360	GII.17[P17]	2022	UK
PQ336859	WT-NORO-2164	GII.17[P17]	2022	UK
PQ336860	WT-NORO-2231	GII.17[P17]	2022	UK
PQ336861	WT-NORO-2271	GII.17[P17]	2022	UK
PQ336862	WT-NORO-2320	GII.17[P17]	2022	UK
PQ336863	WT-NORO-2197	GII.17[P17]	2022	UK
PQ336864	WT-NORO-2306	GII.17[P17]	2021	UK
PQ336865	WT-NORO-2196	GII.17[P17]	2022	UK
PQ336866	WT-NORO-2198	GII.17[P17]	2022	UK
PQ336867	WT-NORO-2116	GII.17[P17]	2022	UK
PQ336868	WT-NORO-2012	GII.17[P17]	2021	UK
PQ336869	WT-NORO-1984	GII.17[P17]	2021	UK
PQ336870	WT-NORO-2273	GII.17[P17]	2022	UK
PQ336871	WT-NORO-2324	GII.17[P17]	2022	UK
PQ336872	WT-NORO-1181	GII.17[P17]	2019	UK
PQ336873	WT-NORO-1506	GII.17[P17]	2019	UK
PQ336942	NL100266	GII.17[P17]	2024	Netherlands
PQ336943	NL100239	GII.17[P17]	2024	Netherlands
PQ336944	NL100277	GII.17[P17]	2024	Netherlands
PQ336945	NL100282	GII.17[P17]	2024	Netherlands
PQ373129	RIVM-NoV22-08	GII.17	2022	Netherlands
PQ373130	RIVM-NoV22-16	GII.17	2022	Netherlands
PQ373131	RIVM-NoV22-22	GII.17	2022	Netherlands
PQ373132	RIVM-NoV22-23	GII.17	2022	Netherlands
PQ373134	RIVM-NoV22-38	GII.17	2022	Netherlands
PQ373135	RIVM-NoV22-45	GII.17	2022	Netherlands
PQ373136	RIVM-NoV22-52	GII.17	2022	Netherlands
PQ373137	RIVM-NoV23-01	GII.17	2023	Netherlands
PQ373138	RIVM-NoV23-37	GII.17	2023	Netherlands
PQ373139	RIVM-NoV23-43	GII.17	2023	Netherlands

PQ373140	RIVM-NoV23-44	GII.17	2023	Netherlands
PQ373141	RIVM-NoV23-50	GII.17	2023	Netherlands
PQ373142	RIVM-NoV24-03	GII.17	2024	Netherlands
PQ373143	RIVM-NoV24-06	GII.17	2024	Netherlands
PQ373144	RIVM-NoV24-09	GII.17	2024	Netherlands
PQ373145	RIVM-NoV24-11	GII.17	2024	Netherlands
PQ373146	RIVM-NoV24-13	GII.17	2024	Netherlands
PQ373147	RIVM-NoV24-14	GII.17	2024	Netherlands

[a]The sequences from the Netherlands are also available through NoroNet.

Figure S1: Patristic and P-distance comparison of GII.P17 P-types including sub-lineage II with their phylogenetically closest GII.P3 and GII.P13 P-types. Error bars represent 2xSD[a] for each P-type comparison. Y-axis represents comparison of distances within and in-between P-types. Below the dotted line, distances within P-type are indicated and above the dotted line, distances between P-types are indicated.

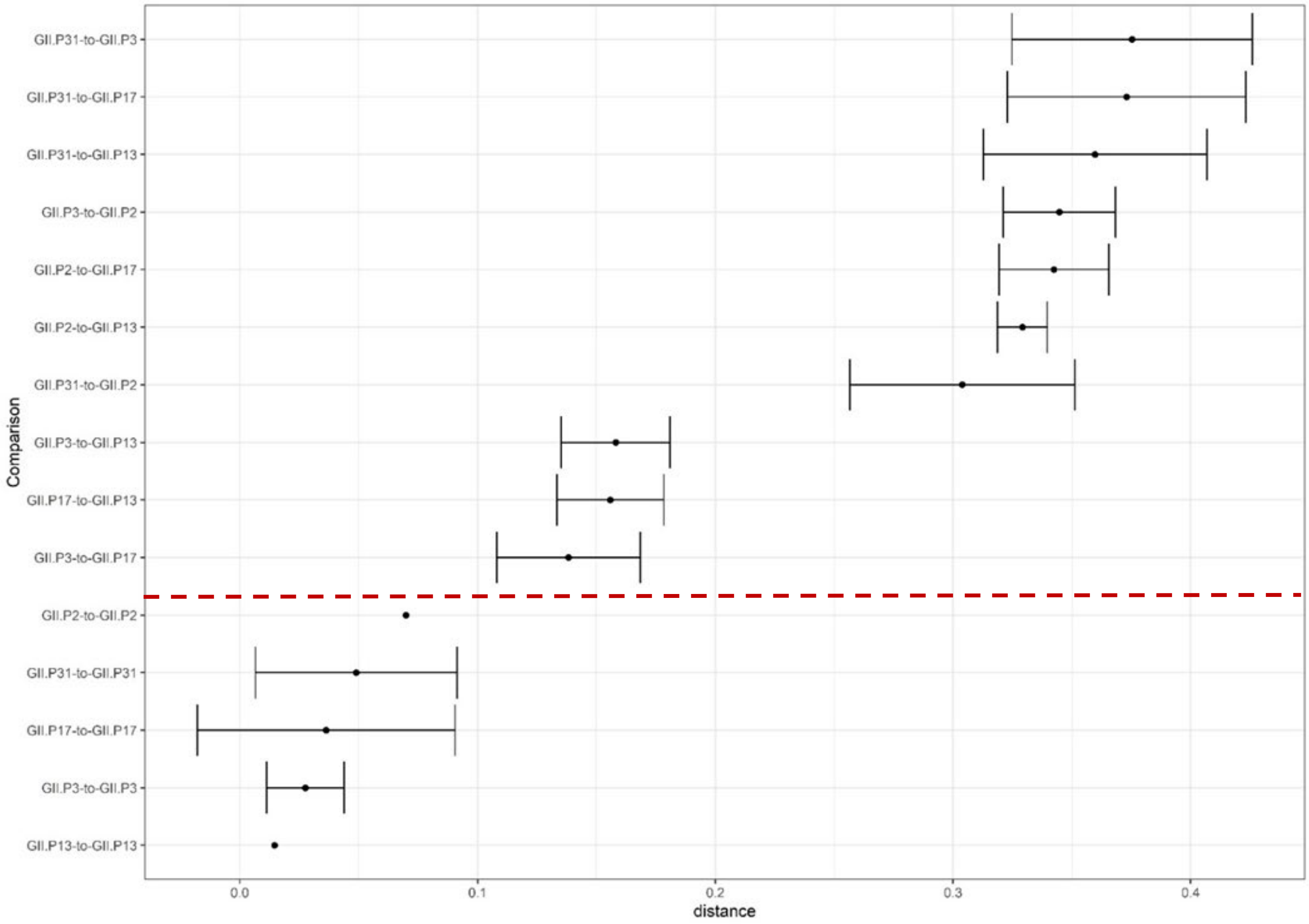

[a]2×SD criteria: phylogenetic distances of sequences within a P-type should not overlap with distances between different P-types [3]

Figure S2: Maximum likelihood phylogenetic trees of norovirus nucleotide sequences from the 5'end of ORF2 was computed within MEGA11 [18] using Tamura-Nei [20] model and 100 bootstrap replications. Known GII.17 variants were identified using the prototype sequences (black). Strains from Ireland are in red.

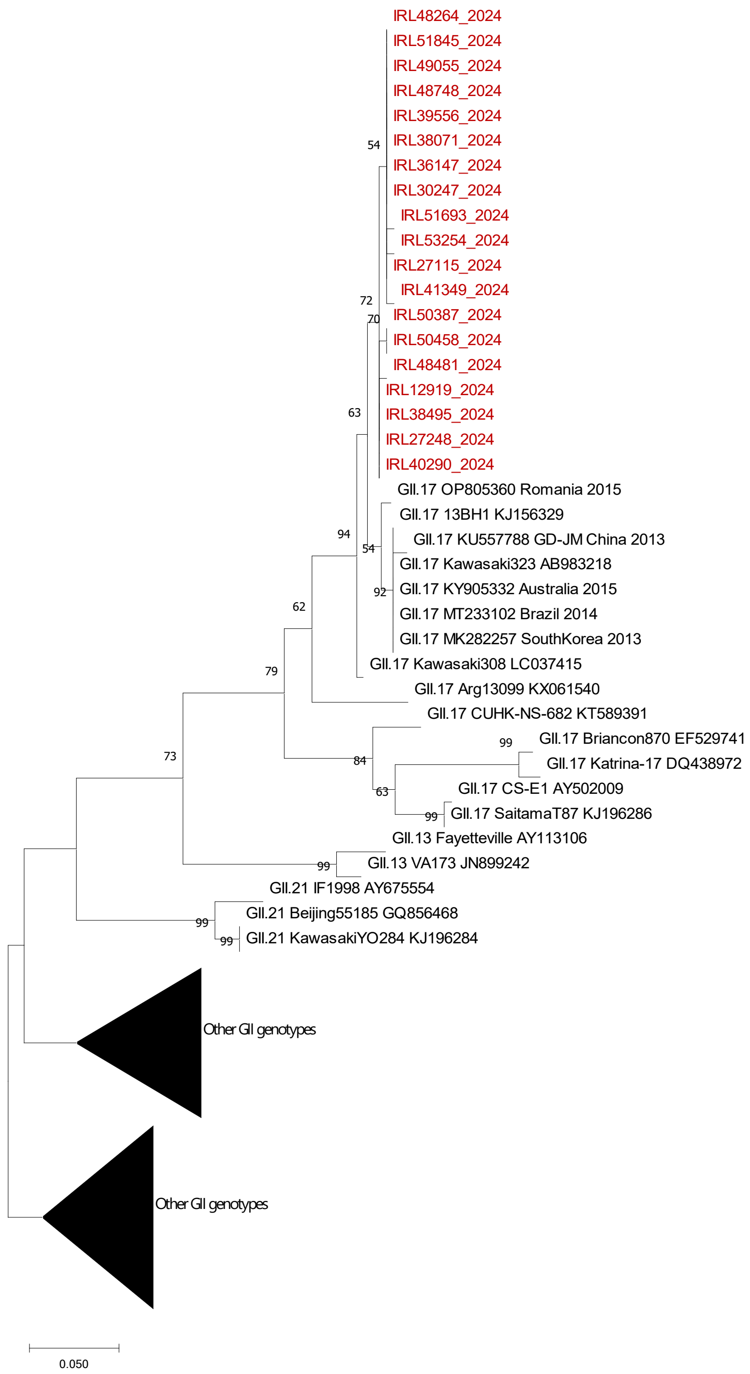

Figure S3: Maximum likelihood phylogenetic trees of norovirus amino acid sequences from the P2 domain of ORF2 was computed within MEGA11 [18] using the Jones-Taylor-Thornton [18] model and 100 bootstrap replications. Known GII.17 variants were identified using the prototype sequences (black). Strains from Austria are in red and Germany in blue.

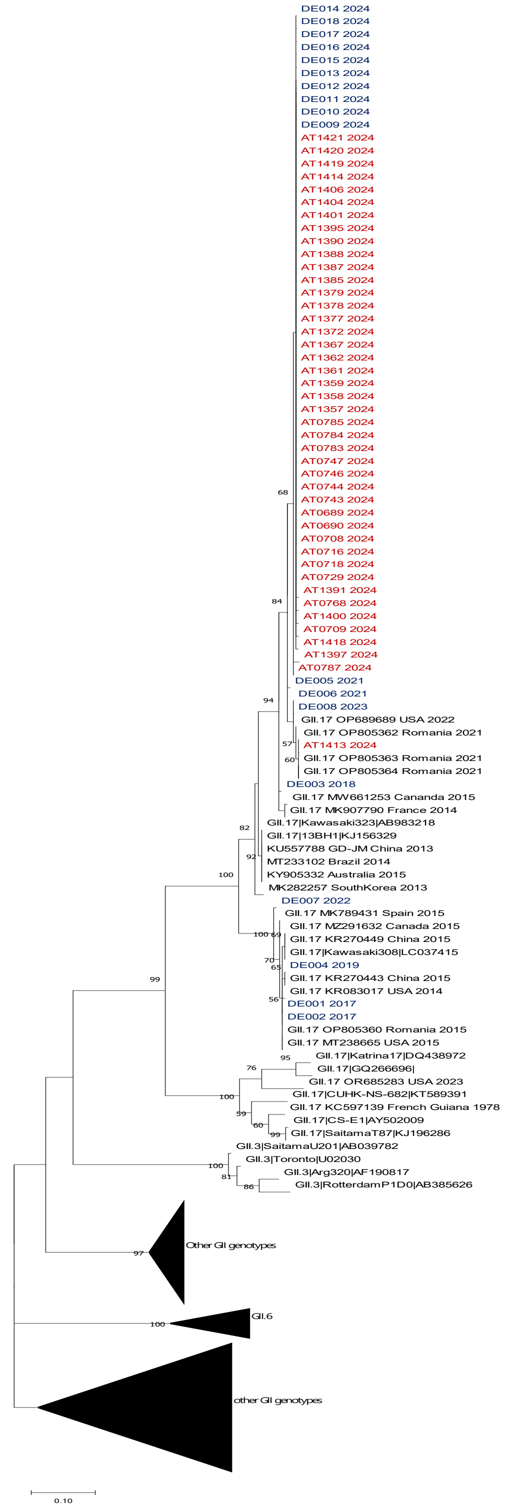

Figure S4: Maximum-likelihood phylogenetic trees of norovirus nucleotide sequences from the 3’ end of ORF1 (RdRp) was computed within MEGA11 [18] using Tamura-Nei [20] model and 100 bootstrap replications. Known GII.17 variants were identified using the prototype sequences (black). Strains from Austria are in red and from Germany in blue.

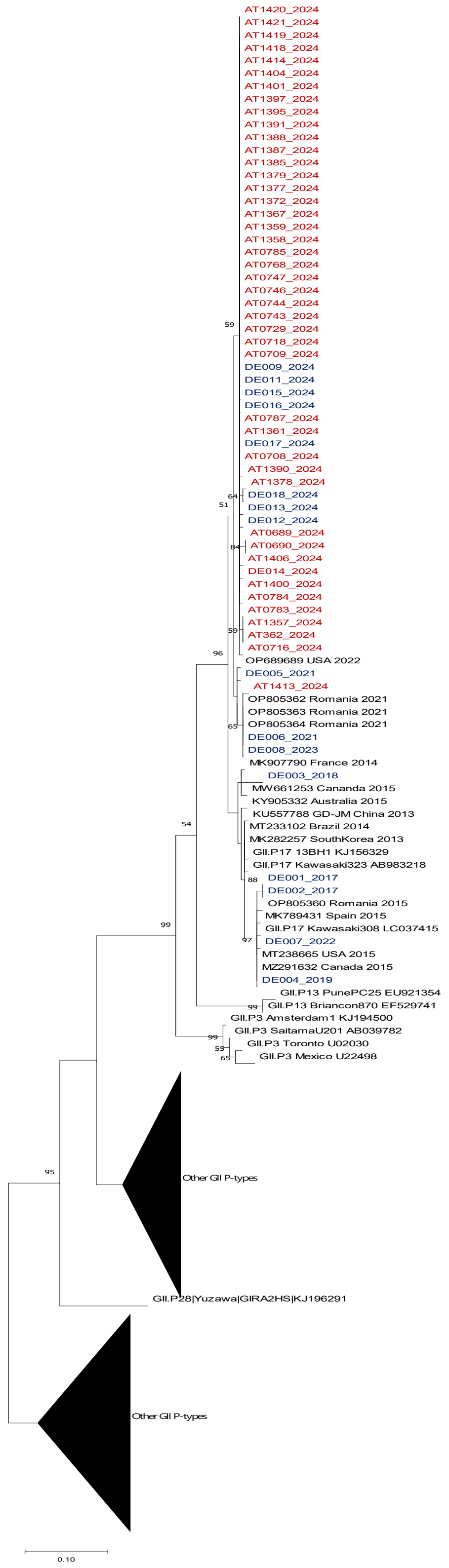

Supplement: Supplement [file 24-00625_DOUGLAS_Supplement.pdf]
